# Prevalence and factors associated with depression among medical students in Cameroon: a cross-sectional study

**DOI:** 10.1186/s12888-017-1382-3

**Published:** 2017-06-09

**Authors:** Stewart Ndutard Ngasa, Carlson-Babila Sama, Bonaventure Suiru Dzekem, Kilton Neba Nforchu, Maxime Tindong, Desmond Aroke, Christian Akem Dimala

**Affiliations:** 1 Galactic Corp Research Group, Buea, Cameroon; 2 Sporedata Research Inc, Durham, North Carolina, USA; 3 Bambalang Medicalised Health Center, Bambalang, Cameroon; 4 Health Services Partner Cameroon, Kumba, Cameroon; 5 Andek Medicalised Health Center, Andek, Cameroon; 6 School of Public Health, Université Libre de Bruxelles (ULB), Brussels, Belgium; 7 Mbengwi District Hospital, Mbengwi, Cameroon; 8 Faculty of Epidemiology and Population Health, London School of Hygiene and Tropical Medicine, London, UK

**Keywords:** Depression, Medical student, Prevalence, Predisposing factors, Self-reported academic performance

## Abstract

**Background:**

Depression is an important contributor to the global burden disease that affects people of communities all over the world. With high level of demands in academics and psychosocial pressure, medical students during their course of training tend to become depressed, leading to problems later in professional life and compromising patient care. In Cameroon, there is lack of data on the prevalence of depression and its impact on medical students. To determine the prevalence and predisposing factors associated with depression among medical students in Cameroon (preclinical and clinical). We also evaluated the impact of depression on self-reported academic performance.

**Methods:**

A cross sectional study was carried out in all 4 state medical schools in 4 different regions from December 2015 to January 2016. Diagnosis of depression, major depression and its associated factors were assessed using the 9-Item-Patient Health Questionnaire (PHQ-9) and a structured questionnaire respectively. We included 618 medical students (response rate: 90.4%).

**Results:**

About a third of them (30.6%, 95% CI: 22.8–36.7) were found to have major depressive disorder (PHQ Score ≥ 10). With regards to the severity of depression, 214 (34.6%), 163 (26.4%), 21 (3.4%), and 5 (0.80%) students were classified as having mild, moderate, moderately severe and severe depression respectively. The presence of a chronic disease (OR: 3.70, 95% CI: 1.72–7.94, *p* = 0.001), major life events (OR: 2.17, 95%CI: 1.32–3.58, *P* = 0.002), female gender (OR: 1.59, 95% CI: 1.06–2.37, *p* = 0.024) and being a student at the clinical level (OR: 4.26, 95% CI: 2.71–6.71, *p* < 0.001) were independently associated with depression. There was no association between depression and self-reported academic performance, (OR: 1.2, 95% CI: 0.9–1.7, *p* = 0.080).

**Conclusion:**

The prevalence of major depressive disorders among medical students in Cameroon is high and is associated with the presence chronic disease, major life events, female gender and being a student at the clinical level. So we recommend clinicians attending to medical students with demographic features suggestive of greater risk of depression, to make an in depth investigation on the possible presence of depression. Despite this high prevalence of major depression among medical students, it was not associated with self-reported academic performance.

## Background

Depression is a mental disorder characterized by loss of interest and pleasure (anhedonia), decreased energy (anergy), feelings of guilt or low self-worth, disturbed sleep and/or appetite, and poor concentration [1]. It is a significant contributor to the global burden of disease and affects people in all countries across the world with a global prevalence of depressive episode of 3.2% [2]. Depressive disorders often start at a young age and often are recurrent throughout life. For these reasons, depression is the leading cause of disability worldwide in terms of total years lost due to disability [2]. The demand for curbing depression and other mental health conditions is thus on the rise globally [2]. Worldwide, it has been demonstrated that 25–90% of medical students are stressed, that is an important determinant of depression [3, 4] leading to a higher prevalence of depression among medical students than general population [5, 6]. Several factors may account for this fact. These include daily life stressors and stressors specific to the tedious learning environment [7]. The potential negative effects of emotional distress on medical students include impairment of functioning in classroom and clinical practice, stress-induced disorders and deteriorating performance. In medical doctors, it has been demonstrated that depression affects patient care leading to increase prescription error [8]. Depression is also associated with higher suicide rates and this may be reason for higher suicide rate in medical professionals than the general population [9]. This is especially true in female medical professionals [10]. Students in extreme stress or depression need serious attention, otherwise inability to cope successfully with the enormous stress of education may lead to a cascade of consequences at both personal and professional levels [11]. To prevent depressive symptoms among medical students, decreased self-esteem, self-perceived medical errors and thus improve on the quality of care given to patients, factors associated with depression in medical training should be identified and appropriately tackled [12]. However, there is scarcity of data in Africa and worse still in Cameroon there is no data on depression and its associated factors among medical students. The aim of this study was to investigate the prevalence and factors associated with depression among preclinical and clinical medical students in Cameroon medical schools and also to evaluate the impact of depression on self-reported academic performance.

## Methods

### Study design, setting and participants

We conducted a cross-sectional study from December 2015 to January 2016 among medical students in Cameroon; a bilingual nation located in Sub-Saharan Africa and has seven medical schools. Four of these schools are public institutions and three are private. Other than training medical doctors, these institutions also train nurses, laboratory scientists, dentists, and pharmacists. Overall, these institutions have over 5000 medical students each academic year. In this study we sampled students from all state medical schools. The four public medical schools include: Faculty of Health Sciences, University of Buea (FHS-UB); Faculty of Health Sciences, University of Bamenda (FHS-UBa); Faculty of Medicine and Pharmaceutical Sciences, University of Douala (FMPS); and Faculty of Medicine and Biomedical Sciences, University of Yaoundé (FMBS). Each of the four public medical schools are located in major cities in 4 different regions of Cameroon and have similar training programs and much lower tuition fees compared to the 3 private medical schools.

### Sample size calculation

The sample size was obtained using the formula for estimation of a proportion [13]

$$ N=\frac{\left[4{\left(\mathrm{Zcrit}\right)}^2\mathrm{P}\left(1-\mathrm{P}\right)\right]}{{\mathrm{D}}^2} $$

Where,

N = Number of participants

Z _crit_ = the standard normal deviate, corresponding to a significance criterion of 0.05 (95), = 1.960

D = Amount of error we will tolerate = +/− 5%

P = Pre-study estimate of the global prevalence of depression among medical students, 33% as reported in a systematic review study [14].

$$ N=\frac{\left[{4(1.960)^2}^{\ast }{0.330}^{\ast }0.670\right]}{(0.1)^2} $$

*N* = 340 medical students were to be included in this study, however to account for sampling error 800 questionnaires were distributed in the different medical schools.

### Sampling and data collection

A cluster sampling method was used where students were recruited by stratified random sampling. Participants were stratified into different medical schools and 200 participants were randomly selected from clinical and preclinical levels of each stratum (each of the four medical schools). Equal numbers were selected from the clinical and preclinical levels. This number represented approximate 25% of the total population. Data were collected on; socio-demographic characteristics, academic/medical history and 9-item patient health questionnaire (PHQ-9). Prior to the commencement of data collection, the questionnaire was pre-tested on 40 students from each stratum. The students who took part in the pre-test were excluded from the study but rather aided in distribution of the questionnaires. All questionnaires which were wrongly filled or had many missing data were not included in the analysis.

### Outcome variables

Our main outcome of interest was provisional diagnosis of depression and it was assessed using the 9-item Patient Health Questionnaire (PHQ-9). The PHQ-9 questionnaire is a self-administered version of the PRIME-MD (Primary Care Evaluation of Mental Disorders) which assesses the presence of major depressive disorder using modified Diagnostic and Statistical Manual fourth edition (DSM-IV) criteria. Using the mental health professional (MHP) re-interview as the criterion standard, a PHQ-9 score ≥ 10 had a sensitivity of 88% and a specificity of 88% for major depression [15]. In addition to making criteria-based diagnoses of depressive disorders, the PHQ-9 is also a reliable and valid measure of depression severity. These characteristics plus its simplicity and brevity make the PHQ-9 a useful clinical and research tool [16]. A provisional diagnosis of depression is made if the PHQ-9 score is greater than 4 and the presence of major depressive disorder if the score is greater than or equal to 10. In this study, ‘depression’ and ‘depressive symptoms are used interchangeably. The severity of depression was classified as follows: mild [5–9], moderate [10–14], moderately severe [15–19] and severe (20–27) depression [15]. Self-reported academic performance was also evaluated. It was evaluated using the grade point average (GPA). This is a number representing the average value of the accumulated final grades earned in all courses over time. A student’s GPA is calculated by adding up all accumulated final grades and dividing that figure by the number of grades. This is graded on a scale ranging from zero to four. In this study, participants were categorized into those with a GPA ≥ 3 and GPA < 3.

### Independent variables

The following variables were evaluated for association with depression: major life event (Participants who had lost a close family member or friend, road traffic accident, rape, break ups and/or hospitalisation for major illness within the previous three months), alcohol consumption (consumption of greater than 21 units/week and greater than 14 units/week for males and females respectively), Chronic illness (accepting to have sickle cell disease, asthma, diabetes or hypertension) and level of study (Preclinical students were students from 1st to 3rd year of medical studies while clinical students made up students from 4th to 7th year).

### Statistical methods

We started our analysis by exploring the distributions, frequencies and percentages for each of the numeric and categorical variables. Data were entered and analysed using Epi info version 7 statistical software. Descriptive analyses were used to summarise data. Results are presented as counts (and percentages for categorical variables), means and standard deviation (SD) or median and interquartile range as appropriate for continuous variables. At bivariate analysis, all independent variable with *p* < 0.05 were selected for multivariate analysis. Multivariate logistic regressions were used to identify independent associations with depressive symptoms and presented as odd ratios (OR) with 95% confidence intervals (CI). A *p*-value <0.05 was set as the threshold of statistical significance.

## Results

### Socio-demographic characteristics of participants

During the study period, a total of 800 questionnaires were distributed to eligible participants of which 723 were returned (response rate: 90.4%). In total, 618 questionnaires were properly filled and analyzed. The participants’ ages ranged from 18 to 28 years with a mean of 22.4 ± 1.9 years. A plurality of the study participants consisted of males (53.7%) and clinical medical students (59.7%). FMPS Douala had the most students (26.4%) within the study sample. A minority (20.1%) of the participants had recent major life events, a few presented with chronic medical disease (6.8%), and some regretted studying medicine (14.2%). More than a third of participants consume alcohol above the cutoff value (38.8%). Majority (69.8%) of the students has had at least a resit examination (69.8%), with just a few (5.5%) ever repeating a year of medical studies. The mean GPA of all participants was 2.9 (1.6 ± 3.6) with 52.9% of participants having a GPA above 3(Table 1)

**Table 1:** Socio-demographic characteristics of participants

Variable	N (618)	Female (286)	Male (332)	*P*-value
Age (Years)	22.43 (+/− 1.94)	22.1 (+/− 1.82)	22.7 (+/−1.99)	< 0.001
Level of studies				< 0.001
Clinical	369 (59.7%)	201 (70.3%)	168 (50.6%)	
Pre-clinical	249 (40.3%)	85 (29.7%)	164 (49.4%)	
Medical School				0.65
FHS-Bamenda	150 (24.3%)	65 (22.7%)	85 (25.6%)	
FHS-Buea	149 (24.1%)	75 (26.2%)	74 (22.3%)	
FMBS-Yaoundé	156 (25.2%)	70 (24.5%)	86 (25.9%)	
FMPS-Douala	163 (26.4%)	76 (26.6%)	87 (26.2%)	
Major life event				0.025
	124 (20.1%)	69 (24.1%)	55 (16.6%)	
Presence of chronic disease				0.207
	42 (6.8%)	15 (5.2%)	27 (8.1%)	
Have regret studying medicine				< 0.001
	87 (14.1%)	24 (8.4%)	63 (19%)	
Alcohol consumption				< 0.001
	240 (38.8%)	85 (29.7%)	155 (46.7%)	
Resit examination				< 0.001
	428 (69.3%)	218 (76.2%)	210 (63.3%)	
Repeat a year of medical studies				0.662
	34 (5.5%)	14 (4.9%)	20 (6%)	
GPA	3.17 (+/−2.68)	3.25 (+/−3.91)	2.93 (+/− 0.37)	0.027

*N* = Total number of participants, *FHS* Faculty of Health Sciences, *FMPS* Faculty of Medical and Pharmaceutical Sciences, *FMBS* Faculty of Medicine and Biomedical Sciences, *GPA* Grade Point Average

### Prevalence of depression

**Fig. 1:**
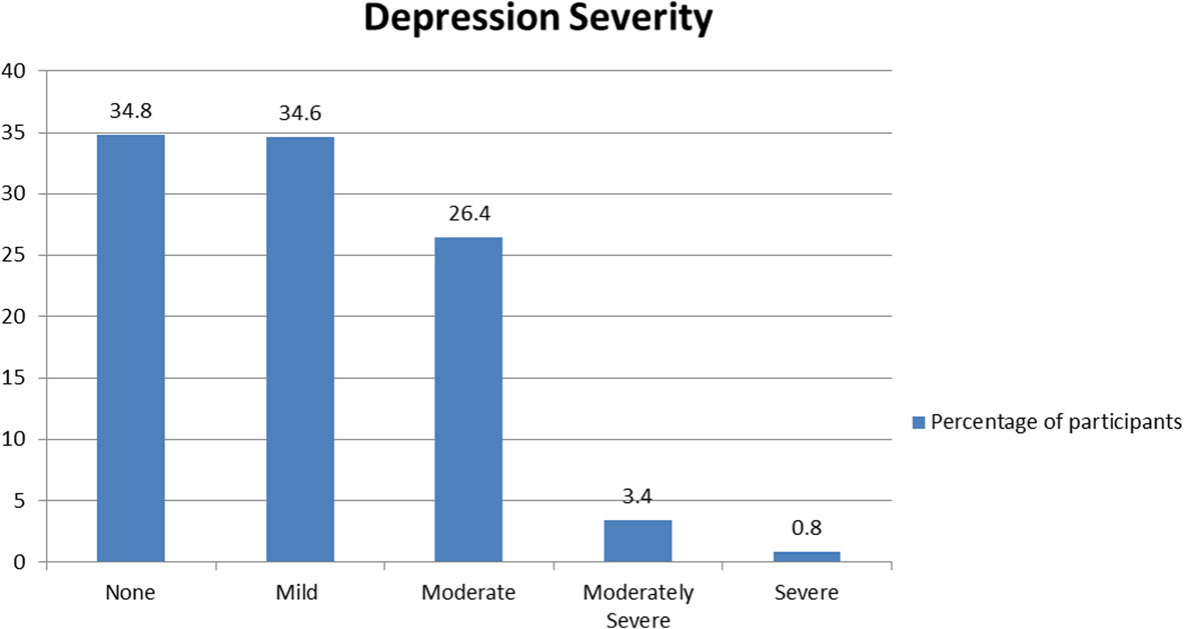
Severity of Depression among medical students in Cameroon

The PHQ-9 score of participants ranged from 0 to 21 with a median score of 6 (interquartile range 3–10). Overall, 403 (65.2%) students were diagnosed with provisional depression (PHQ ≥ 4) while 189 had a PHQ-9 score of ≥10, giving a prevalence of 30.6% (95% CI: 22.8 ± 36.7) for major depressive disorder. Among the 403 participants provisionally found to have a depressive disorder, 214 (34.6%); 163 (26.4%); 21 (3.4%); and 5 (0.80%) of them were found to have symptoms of mild, moderate, moderately severe and severe depression respectively (Fig. 1).

### Prevalence of depression across the different medical schools

**Fig. 2:**
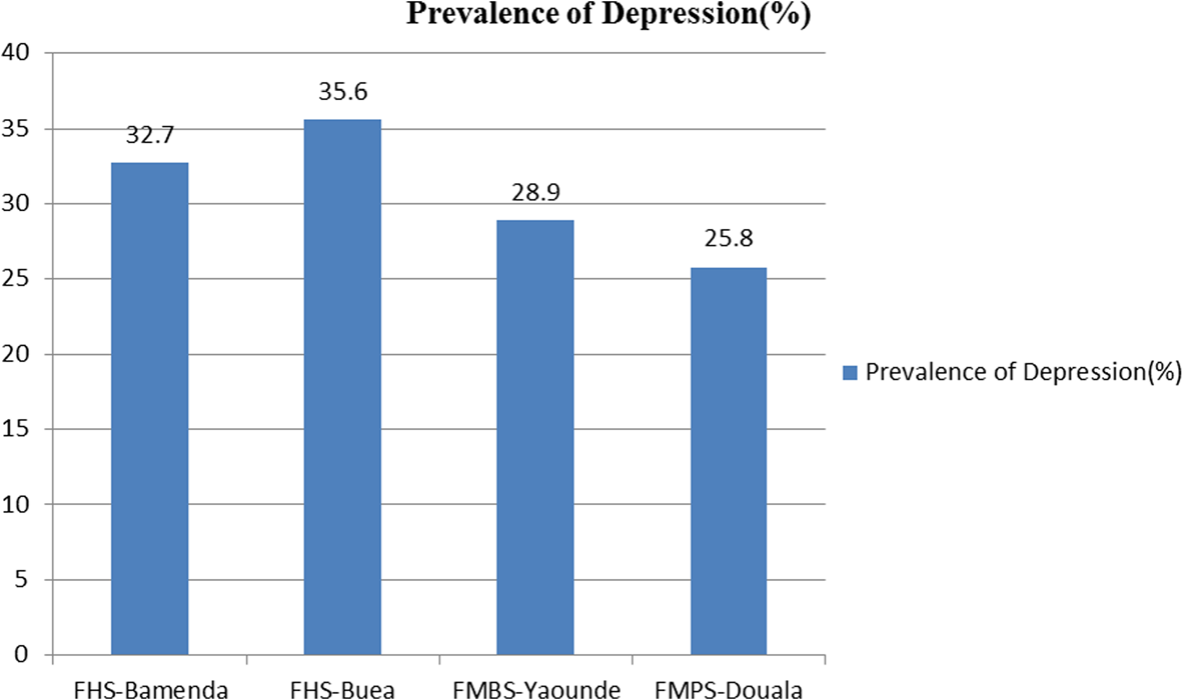
Prevalence of depressive symptoms in the four public medical schools in Cameroon

The prevalence of depression was highest in the FHS-UB (35.6%) while it was least in FMPS Douala (25.8%) (Fig. 2). The difference in the prevalence of depression among the various medical schools was not significant (*p* = 0.26)

### Factors associated with depression among medical students

At bivariate analysis, regret studying medicine (*p* < 0.001), presence of chronic disease (*p* < 0.001), presence of a recent major life event (*p* = 0.018), female sex (*p* = 0.011) and being a student at the clinical level (*p* = 0.001) were significantly associated with depression. In multivariate analysis, the presence of a chronic disease (*p* = 0.001), major life events (0.002), female sex (*p* = 0.024) and being a student at the clinical level (*p* < 0.001) were independently associated with depression (Table 2).

**Table 2:** Factors associated with depression

Variable		Crude ORs (CI)	*p*-value	Adjusted ORs (CI)	*p*-value
Age (years)	18–22	0.69 (0.45–1.06)	0.11		
	23–28	1			
Gender	Female	1.56 (1.11–2.20)	0.011	1.59 (1.06–2.37)	0.024
	Male	1			
Level of study	Clinical	4.38 (2.90–6.62)	<0.001	4.26 (2.71–6.71)	<0.001
	Preclinical	1			
Major life event	Yes	1.75 (1.1–2.79)	0.018	2.17 (1.32–3.58)	0.002
	No	1			
Chronic diseases	Yes	5.16 (2.65–10.04)	<0.001	3.70 (1.72–7.94)	0.001
	No	1			
Regret studying medicine	Yes	2.28 (1.43–3.61)	<0.001	1.64 (0.96–2.78)	0.069
	No	1			
Alcohol consumption	Yes	1.12 (0.78–1.59)	0.54		
	No	1			
Resit	Yes	1.41 (0.97–2.03)	0.070	2.06 (1.35–3.16)	0.001
	No	1			

*OR* Odds ratio, *CI* Confidence interval

### Depression and self-reported academic performance

There was no association between depression and self-reported academic performance (GPA), OR: 1.2 (0.9–1.7) and *p*-value = 0.08 (Table 3).

**Table 3:** Depression and self-reported academic performance

	Depression (%)		OR (CI)	*p*-value
GPA ≥ 3 (%)	No	Yes	1.4 (0.9–2.O)	0.08
Yes	220 (35.6)	112 (18.1)		
No	209 (33.8)	77 (12.4)		
Total	429 (69.4)	189 (30.6)		

*OR* Odds ratio, *CI* Confidence interval

## Discussion

To the best of our knowledge this is the first study in Cameroon and the Central African Sub-region to determine the prevalence and factors associated with depressive symptoms among medical students. We also investigated the association between depressive symptoms and self-reported academic performance. In our study about one out of every three medical student is depressed with a considerable number being moderate to severely depressed. Depressive symptoms were significantly associated with chronic disease, major life events, female gender and being a student at the clinical level. However no association was found between depression and self-reported academic performance. Medical training programmes aim to produce knowledgeable, skillful, competent and professional graduates who will render comprehensive healthcare services within their communities. To achieve this aim, most medical programmes are overloaded with facts. This may have unintended negative consequences with respect to students personal mental and physical health [7]. In our study the prevalence of depression was 30.6% and was similar to the global prevalence of 33.0% among university students reported in a systematic review study [14]. Our prevalence was higher than the prevalence of 23.3% reported in a study among undergraduate medical students in Nigeria [17]. In their study, they used a single medical school with a smaller study population. Out of Africa, the variation in the prevalence of depression is even more significant with systematic review studies showing prevalences ranging from 6.0 to 66.5% [18]. Despite this variation in prevalences of depression around the world, studies have consistently showed high prevalence of depression in medical students. Several factors can be advanced for this variation. These include differences in the lengths of training programs, cost of studies, the use of different depression scoring tools and cultural differences around the world [19]. In our study, majority of students presented with mild depression, several presented moderate depression while many presented with moderately severe to severe depression. These findings concur with findings in a study by Kumar and colleagues where 29.8% of participants were scored as normal while 27.8% as mild, 29.3% as moderate, 7.5% as severe, and 6.7% as very severe depression [20]. With regards to the four medical schools included in this study, the prevalence of depression was highest among medical students of the FHS-UB and lowest in the FMPS. This difference was not statistically significant and may be due to the fact that these schools have similar study programs. A higher prevalence was observed in clinical students than pre-clinical students. Previous studies have shown depressive symptoms is not only higher in students in higher levels of studies but also increases as students progress in medical schools [20]. Furthermore depression was significantly associated with the female gender. This fact was supported in study by Dahlin and colleagues in 2005 [21], while other studies have demonstrated marginal increases in depression in female medical students compared to males [20]. Other studies have also reported significant association between depression and the presence of chronic medical conditions and major life events [22]. However, these two are also differential diagnoses of depression. Unlike in our study where depression was not associated with self-reported academic performance, significant association has been reported between depression and self-reported academic performance [22, 23]. In these studies, it was found that depressed students were more likely to have a GPA < 3 than those not depressed. This could be explained by the fact that students with depressive symptoms are less motivated to study or even take care of their patients. This ultimately leads to poor performance.

Despite filling an important gap in the literature, our study does have limitations that are usually associated with an observational design. First, recall bias is limitation of this study as during the filling of the questionnaire some participants may have difficulty recollecting some information leading to inaccuracy in the data entered. However, the sufficient sample size and using a valid score like the PHQ-9 scale to diagnose depression and depressive symptoms of the students increases the validity of the study. Also, when evaluating alcohol consumption we used the cutoff of 21 units/week for men and 14 units/week for women. This cut off is mostly referred to as the limits of low risk drinking. This recommendation was recently changed in January 2016 by Department of Health UK to 14 units/week for both men and women [24].

## Conclusion

Depression among medical students in Cameroon is high and is associated with chronic medical condition, major life events, female gender and being a student at the clinical level. The prevalence of provisional depressive disorder and major depressive disorder among medical students was high. Many students presented with moderate to moderately severe depression while a few presented with severe depression.

### Implication

We recommend the creation of support groups with counseling facilities within medical schools in Cameroon. We will also recommend clinicians who receive students with such demographics and therefore at a greater risk of having depressive symptoms to make an in depth investigation on the presence on depression in these patients and engage on a holistic management of their condition. Furthermore, there should be routine screening for depression in medical students with major life events, female medical students, clinical level students and students with chronic medical disease with subsequent appropriate management. Depression was not associated with academic performance; however we recommend further studies using other depression scoring tools to further explore these findings in Cameroon.

## Abbreviations

CI: Confidence Interval
DSM-IV: Diagnostic and Statistical Manual fourth edition
FHS-UB: Faculty of Health Sciences, University of Buea
FHS-UBa: Faculty of Health Sciences, University of Bamenda
FMBS: Faculty of Medicine and Biomedical Sciences, University of Yaoundé
FMPS: Faculty of Medicine and Pharmaceutical Sciences University of Douala
GPA: Grade Point Average
MHP: Mental Health Professional
OR: Odds Ratio
PHQ-9: Patient Health Questionnaire
PRIME-MD: Primary Care Evaluation of Mental Disorders
SD: Standard Deviation

## References

[1] Marcus M, Yasamy MT, van Ommeren M, Chisholm D, Saxena S, others. Depression: a global public health concern. WHO Department of Mental Health and Substance Abuse 2012;1:6–8.

[2] Moussavi S, Chatterji S, Verdes E, Tandon A, Patel V, Ustun B (2007). Depression, chronic diseases, and decrements in health: Results from the world health surveys. Lancet (London, England).

[3] Koochaki GM, Charkazi A, Hasanzadeh A, Saedani M, Qorbani M, Marjani A. Prevalence of stress among iranian medical students: A questionnaire survey. Eastern Mediterranean health journal = La revue de santeÌ de la MeÌ diterraneÌ e orientale = al-Majallah al-sÌ£ihÌ£hÌ£iÌ„yah li-sharq al-mutawassitÌ£. 2011 Jul;17(7):593–8. 21972483

[4] Sherina MS, Rampal L, Kaneson N (2004). Psychological stress among undergraduate medical students. Med J Malaysia.

[5] Dyrbye LN, Thomas MR, Shanafelt TD (2006). Systematic review of depression, anxiety, and other indicators of psychological distress among u.S. and canadian medical students. Academic medicine : journal of the Association of American Medical Colleges.

[6] Bramness JG, Fixdal TC, Vaglum P (1991). Effect of medical school stress on the mental health of medical students in early and late clinical curriculum. Acta Psychiatr Scand.

[7] Naidoo S, Van Wyk J, Higgins-Opitz SB, Moodley K. An evaluation of stress in medical students at a south african university. South African Family Practice. Taylor & Francis; 2014;56(5):258–62.

[8] Fahrenkopf AM, Sectish TC, Barger LK, Sharek PJ, Lewin D, Chiang VW (2008). Rates of medication errors among depressed and burnt out residents: prospective cohort study. BMJ (Clinical research ed).

[9] Frank E, Biola H, Burnett CA (2000). Mortality rates and causes among u.S. physicians. Am J Prev Med.

[10] Lindeman S, Laara E, Hakko H, Lonnqvist J (1996). A systematic review on gender-specific suicide mortality in medical doctors. The British journal of psychiatry : the journal of mental science.

[11] Ibrahim MB, Abdelreheem MH (2015). Prevalence of anxiety and depression among medical and pharmaceutical students in alexandria university. Alexandria Journal of Medicine Elsevier.

[12] Iqbal S, Gupta S, Venkatarao E (2015). Stress, anxiety & depression among medical undergraduate students & their socio-demographic correlates. Indian J Med Res.

[13] Eng J (2003). Sample size estimation: how many individuals should be studied? 1. Radiology Radiological Society of North America.

[14] Sarokhani D, Delpisheh A, Veisani Y, Sarokhani MT, Manesh RE, Sayehmiri K. Prevalence of depression among university students: A systematic review and meta-analysis study. Depression research and treatment. 2013;2013:373857. 10.1155/2013/373857 PMC3800630 24187615

[15] Kroenke K, Spitzer RL, Williams JB (2001). The pHQ-9: validity of a brief depression severity measure. J Gen Intern Med.

[16] Kroenke K, Spitzer RL (2002). The pHQ-9: a new depression diagnostic and severity measure. Psychiatric annals SLACK Incorporated.

[17] Aniebue PN, Onyema GO (2008). Prevalence of depressive symptoms among nigerian medical undergraduates. Trop Dr.

[18] Hope V, Henderson M (2014). Medical student depression, anxiety and distress outside north america: a systematic review. Med Educ.

[19] Haroz EE, Bolton P, Gross A, Chan KS, Michalopoulos L, Bass J (2016). Depression symptoms across cultures: an iRT analysis of standard depression symptoms using data from eight countries. Soc Psychiatry Psychiatr Epidemiol.

[20] Kumar GS, Jain A, Hegde S (2012). Prevalence of depression and its associated factors using beck depression inventory among students of a medical college in karnataka. Indian J Psychiatry.

[21] Dahlin M, Joneborg N, Runeson B (2005). Stress and depression among medical students: a cross-sectional study. Med Educ.

[22] Sidana S, Kishore J, Ghosh V, Gulati D, Jiloha R, Anand T (2012). Prevalence of depression in students of a medical college in new delhi: a cross-sectional study. Australas Med J.

[23] Supe AN (2000). A study of stress in medical students at seth g.S. Medical college. J Postgrad Med.

[24] UK Department of Health. Alcohol consumption: Advice on low risk drinking. 2016. https://www.gov.uk/government/publications/alcohol-consumption-advice-on-low-risk-drinking

